# *trans*-Bis(5-amino-1,3,4-thia­diazol-2-thio­lato-κ*S* ^2^)bis­(triphenyl­phosphane-κ*P*)palladium(II) dimethyl sulfoxide disolvate hemihydrate

**DOI:** 10.1107/S1600536812011555

**Published:** 2012-03-24

**Authors:** Felipe Chontal-Vidal, Maricela Arroyo-Gómez, Simón Hernández-Ortega, Reyna Reyes-Martínez, David Morales-Morales

**Affiliations:** a Instituto de Química, Universidad Nacional Autónoma de México, Circuito exterior, Ciudad Universitaria, México, DF 04510, Mexico; b Ciencias Básicas e Ingeniería, Recursos de la Tierra, Universidad Autónoma, Metropolitana. Av. Hidalgo Poniente, La Estación Lerma, Lerma de Villada, Estado de México, CP 52006, Mexico

## Abstract

The title complex, [Pd(C_2_H_2_N_3_S_2_)_2_(C_18_H_15_P)_2_]·2C_2_H_6_OS·0.5H_2_O, was obtained from the reaction of *trans*-[(Ph_3_P)_2_PdCl_2_] with 5-amino-1,3,4-thia­diazole-2-thione (SSNH_2_) in a 2:1 molar ratio. The Pd^II^ atom, located in a crystallographic center of symmetry, has a square-planar geometry with two triphenyl­phosphine *P*-coordinated mol­ecules and two SSNH_2_ ligands with the S atoms in a *trans* conformation. The latter ligand exhibits N—H⋯N hydrogen-bonding contacts formed by the amino group with the thia­diazole ring, generating a chain along the *c* axis. The asymmetric unit contains one half of the complex mol­ecule along with disordered dimethyl sulfoxide and water mol­ecules.

## Related literature

For background to the design and synthesis of ligands that contain efficient metal coordination sites and hydrogen-bonding functionalities, see: Beatty (2001 ▶). The SSNH_2_ (5-amino-1,3,4-thia­diazole-2-thiol) ligand exists in the thione and thiol forms and can converted into the thiol­ate form depending on the affinity of the metal, see: Tzeng *et al.* (1999 ▶). For SSNH_2_ acting as a ligand and as auxiliary in the construction of hydrogen bonds in coordination compounds with Pd^II^, see: Tzeng, Lee *et al.* (2004 ▶), with Pt^II^, see: Tannai *et al.* (2006 ▶), with Cd^II^, see: Gao *et al.* (2009 ▶) and with Au^I^, see: Tzeng *et al.* (1999 ▶); Tzeng, Huang *et al.* (2004 ▶). For the thiol­ate form, see: Downie *et al.* (1972 ▶). 
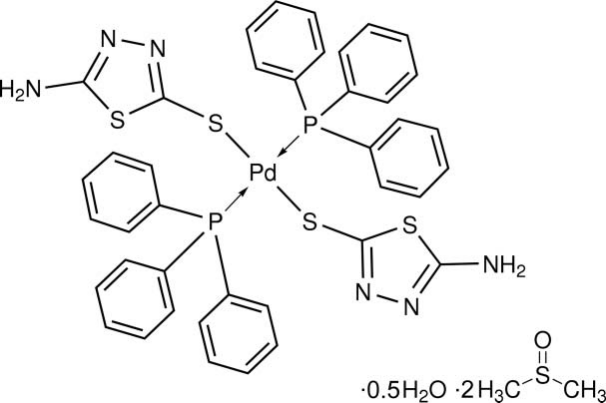


## Experimental

### 

#### Crystal data

[Pd(C_2_H_2_N_3_S_2_)_2_(C_18_H_15_P)_2_]·2C_2_H_6_OS·0.5H_2_O
*M* *_r_* = 1060.58
Orthorhombic, 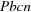

*a* = 14.6192 (18) Å
*b* = 13.2572 (16) Å
*c* = 25.707 (3) Å
*V* = 4982.3 (10) Å^3^
*Z* = 4
Mo *K*α radiation
μ = 0.73 mm^−1^
*T* = 298 K
0.24 × 0.16 × 0.13 mm

#### Data collection

Bruker SMART APEX CCD area-detector diffractometer
38822 measured reflections
4590 independent reflections
2603 reflections with *I* > 2σ(*I*)
*R*_int_ = 0.107

#### Refinement

*R*[*F* ^2^ > 2σ(*F* ^2^)] = 0.057
*wR*(*F* ^2^) = 0.137
*S* = 0.95
4590 reflections
322 parameters
99 restraints
H atoms treated by a mixture of independent and constrained refinement
Δρ_max_ = 0.63 e Å^−3^
Δρ_min_ = −0.37 e Å^−3^

### 

Data collection: *SMART* (Bruker, 2007 ▶); cell refinement: *SAINT* (Bruker, 2007 ▶); data reduction: *SAINT*; program(s) used to solve structure: *SHELXTL* (Sheldrick, 2008 ▶); program(s) used to refine structure: *SHELXTL*; molecular graphics: *SHELXTL*; software used to prepare material for publication: *SHELXTL*.

## Supplementary Material

Crystal structure: contains datablock(s) I, global. DOI: 10.1107/S1600536812011555/br2190sup1.cif

Structure factors: contains datablock(s) I. DOI: 10.1107/S1600536812011555/br2190Isup2.hkl

Additional supplementary materials:  crystallographic information; 3D view; checkCIF report

## Figures and Tables

**Table 1:** Selected bond lengths (Å)

Pd—P1	2.3364 (15)
Pd—S2	2.3407 (14)
S2—C2	1.736 (5)

**Table 2:** Hydrogen-bond geometry (Å, °)

*D*—H⋯*A*	*D*—H	H⋯*A*	*D*⋯*A*	*D*—H⋯*A*
N6—H6*A*⋯N4^i^	0.90 (1)	2.12 (2)	2.986 (7)	160 (5)

Symmetry code: (i) 
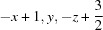
.

**Fig. 1.**
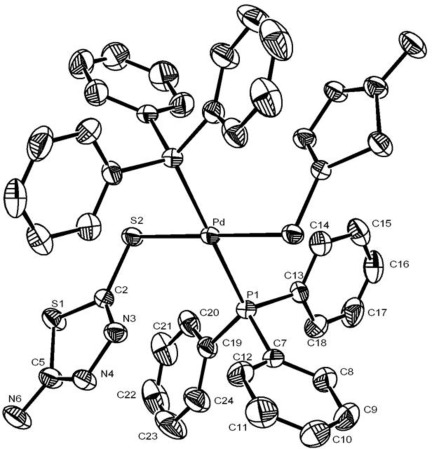
The molecular structure of the title compound with displacement ellipsoids at the 30% probability, the hydrogen atoms, DMSO and H2O solvent are omitted for clarity.

**Fig. 2.**
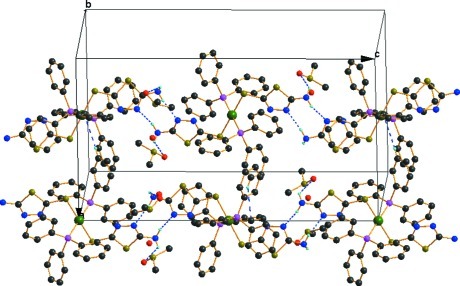
The title compound is linked by N—H···N intermolecular interactions along the c axes, the hydrogen atoms for the interactions are drawn.

## supplementary crystallographic information

### Comment

Hydrogen bonds are commonly used to generate supramolecular assemblies of coordination complexes, in this field an important area of research is the design and synthesis of ligands that contain efficient metal coordination sites and hydrogen bonding functionalities (Beatty, 2001). In this context the ligand 5-amino-1,3,4-thiadiazole-2-thiol (SSNH_2_) has been used as building block for the construction of hydrogen bonded frameworks. The ligand SSNH_2_ can exists in the thione and thiol forms, however it can converted into the thiolate form depending on the affinity of the metal (Tzeng, *et al.*, 1999). Several reports of SSNH_2_ acting as a ligand and as auxiliary in the construction of hydrogen bonds in coordination compounds with Pd(II) (Tzeng, Lee *et al.*, 2004), Pt(II) (Tannai, *et al.*, 2006), Cd(II) (Gao, *et al.*, 2009) and Au(I) (Tzeng, *et al.*, 1999; Tzeng, Huang *et al.*, 2004) have been informed in the literature. Thus, in this opportunity we would like to report the crystal structure of the Pd(II) complex, *trans*-[(Ph_3_P)_2_Pd(SSNH_2_)_2_] DMSO, H_2_O.

The molecular structure of the title compound is shown in Figure 1. The selected bond distances and angles are listed in Table 1. Only half molecule of the complex is found in the asymmetric unit and an inversion operator is needed for the generation of a whole molecule. The Pd(II) atom in the complex exhibits a square-planar arrangement, however the geometry is forced by the steric hindrance and electronic repulsions due to the interactions between the phenyl and the heterocycle rings. The SSNH_2_ ligands are bonded to the metal center by the sulfur atoms in a *trans* arrangement with the thiadiazole groups found out of the plane of the Pd(II) coordination environment. The distance C2-S2 confirms that the ligand exists in the thiolate form (Downie, *et al.*, 1972). The free amine group of the ligand SSNH_2_ forms a hydrogen bond N6—H6A···N4 with the nitrogen atom of the thiadiazole ring related by symmetry, generating a centrosymmetric eight-member cycle, that is extended along the *c*-axes to form a chain framework. These chains are kept together by weak C—H···π [C9–H9···Cg(C13–C18)] intermolecular interactions. The compound crystallized with one molecule of DMSO that exhibits disorder on its structure, and one molecule of water. Weak interactions of N6—H6B···O1 (DMSO) solvent and O2—H atom of the DMSO solvent are observed. Although the solvent molecules do not participate in the strong interactions, they are important in the stabilization of the compound in the crystal lattice.

### Experimental

To a CH_2_Cl_2_ solution (20 ml) of *trans*-[(Ph_3_P)_2_PdCl_2_] (50 mg, 0.07 mmol) a solution of 5-amino-1,3,4-thiadiazole-2-thiol (20 mg, 15 mmol) and triethylamine (2 ml) in CH_2_Cl_2_ (20 ml) was added dropwise, and immediate change from yellow to orange was noted and the resulting reaction mixture was allowed to proceed overnight at room temperature under stirring. After this time, a reddish-orangey precipitated was noted, and the solution was filtered under vacuum to afford compound *trans*-[(Ph_3_P)_2_Pd(SSNH_2_)_2_] (59 mg, 95% yield). Crystals suitable for single-crystal X-ray diffraction analysis were obtained from DMSO/*^i^*PrOH.

### Refinement

H atoms on N were located on the Fourier map and refined isotropically (N—H = 0.90 Å). All H atoms were included in calculated positions (C—H = 0.93 Å), and refined using a riding model with *U*_iso_ (H) = 1.2*U*_eq_ of the carrier atom. The DMSO solvent is disordered and was refined in two major positions using a free variable of Site Occupational Factor (SOF). The ratio of disordered atoms was 55/45 of SOF. O of H_2_O molecule is in crystallographic center of symmetry and its H atom (H_2_O) was not possible to locate on the fourier map.

### Crystal data

**Table:**

[Pd(C_2_H_2_N_3_S_2_)_2_(C_18_H_15_P)_2_]·2C_2_H_6_OS·0.5H_2_O	*F*(000) = 2176
*M**_r_* = 1060.58	*D*_x_ = 1.414 Mg m^−^^3^
Orthorhombic, *P**b**c**n*	Mo *K*α radiation, λ = 0.71073 Å
Hall symbol: -P 2n 2ab	Cell parameters from 6520 reflections
*a* = 14.6192 (18) Å	θ = 2.2–25.0°
*b* = 13.2572 (16) Å	µ = 0.73 mm^−^^1^
*c* = 25.707 (3) Å	*T* = 298 K
*V* = 4982.3 (10) Å^3^	Prism, orange
*Z* = 4	0.24 × 0.16 × 0.13 mm

### Data collection

**Table:**

Bruker SMART APEX CCD area-detector diffractometer	2603 reflections with *I* > 2σ(*I*)
Radiation source: fine-focus sealed tube	*R*_int_ = 0.107
Graphite monochromator	θ_max_ = 25.5°, θ_min_ = 2.1°
Detector resolution: 0.83 pixels mm^-1^	*h* = −17→17
ω scans	*k* = −16→15
38822 measured reflections	*l* = −31→30
4590 independent reflections	

### Refinement

**Table:**

Refinement on *F*^2^	Primary atom site location: structure-invariant direct methods
Least-squares matrix: full	Secondary atom site location: difference Fourier map
*R*[*F*^2^ > 2σ(*F*^2^)] = 0.057	Hydrogen site location: inferred from neighbouring sites
*wR*(*F*^2^) = 0.137	H atoms treated by a mixture of independent and constrained refinement
*S* = 0.95	*w* = 1/[σ^2^(*F*_o_^2^) + (0.0611*P*)^2^] where *P* = (*F*_o_^2^ + 2*F*_c_^2^)/3
4590 reflections	(Δ/σ)_max_ = 0.001
322 parameters	Δρ_max_ = 0.63 e Å^−^^3^
99 restraints	Δρ_min_ = −0.37 e Å^−^^3^

### Special details

**Table:**

Geometry. All e.s.d.'s (except the e.s.d. in the dihedral angle between two l.s. planes) are estimated using the full covariance matrix. The cell e.s.d.'s are taken into account individually in the estimation of e.s.d.'s in distances, angles and torsion angles; correlations between e.s.d.'s in cell parameters are only used when they are defined by crystal symmetry. An approximate (isotropic) treatment of cell e.s.d.'s is used for estimating e.s.d.'s involving l.s. planes.
Refinement. Refinement of *F*^2^ against ALL reflections. The weighted *R*-factor *wR* and goodness of fit *S* are based on *F*^2^, conventional *R*-factors *R* are based on *F*, with *F* set to zero for negative *F*^2^. The threshold expression of *F*^2^ > σ(*F*^2^) is used only for calculating *R*-factors(gt) *etc*. and is not relevant to the choice of reflections for refinement. *R*-factors based on *F*^2^ are statistically about twice as large as those based on *F*, and *R*- factors based on ALL data will be even larger.

### Fractional atomic coordinates and isotropic or equivalent isotropic displacement parameters (Å^2^)

**Table:**

	*x*	*y*	*z*	*U*_iso_*/*U*_eq_	Occ. (<1)
Pd	0.5000	0.5000	0.5000	0.04092 (19)	
S1	0.30706 (10)	0.43464 (14)	0.65323 (6)	0.0614 (5)	
S2	0.35991 (9)	0.47537 (12)	0.54222 (5)	0.0494 (4)	
C2	0.3886 (4)	0.4746 (4)	0.6078 (2)	0.0428 (14)	
N3	0.4650 (3)	0.5019 (4)	0.62829 (17)	0.0527 (13)	
N4	0.4655 (3)	0.4920 (4)	0.68230 (17)	0.0526 (13)	
C5	0.3896 (4)	0.4590 (5)	0.7008 (2)	0.0561 (16)	
N6	0.3726 (4)	0.4426 (5)	0.75160 (19)	0.0872 (19)	
H6A	0.4270 (11)	0.444 (4)	0.7682 (6)	0.105*	
H6B	0.331 (3)	0.489 (3)	0.7616 (8)	0.105*	
P1	0.56548 (10)	0.35283 (12)	0.53336 (5)	0.0469 (4)	
C7	0.6788 (4)	0.3827 (5)	0.5585 (2)	0.0524 (16)	
C8	0.7562 (5)	0.3304 (6)	0.5456 (3)	0.084 (2)	
H8	0.7521	0.2735	0.5246	0.101*	
C9	0.8418 (5)	0.3621 (8)	0.5640 (3)	0.107 (3)	
H9	0.8943	0.3276	0.5541	0.128*	
C10	0.8482 (6)	0.4410 (8)	0.5954 (3)	0.105 (3)	
H10	0.9050	0.4603	0.6083	0.126*	
C11	0.7727 (5)	0.4936 (7)	0.6086 (3)	0.098 (3)	
H11	0.7784	0.5496	0.6301	0.117*	
C12	0.6866 (5)	0.4658 (6)	0.5906 (3)	0.073 (2)	
H12	0.6351	0.5026	0.6000	0.088*	
C13	0.5809 (4)	0.2545 (5)	0.4849 (2)	0.0536 (16)	
C14	0.5676 (4)	0.2755 (5)	0.4331 (2)	0.0606 (17)	
H14	0.5515	0.3401	0.4225	0.073*	
C15	0.5787 (5)	0.1981 (6)	0.3965 (3)	0.076 (2)	
H15	0.5712	0.2122	0.3613	0.092*	
C16	0.5999 (5)	0.1033 (6)	0.4115 (4)	0.081 (2)	
H16	0.6077	0.0531	0.3867	0.098*	
C17	0.6099 (5)	0.0812 (6)	0.4624 (4)	0.092 (3)	
H17	0.6226	0.0153	0.4726	0.110*	
C18	0.6013 (5)	0.1553 (5)	0.4991 (3)	0.077 (2)	
H18	0.6092	0.1395	0.5340	0.092*	
C19	0.5104 (5)	0.2807 (4)	0.5842 (2)	0.0555 (16)	
C20	0.4262 (5)	0.2343 (5)	0.5738 (3)	0.068 (2)	
H20	0.3986	0.2451	0.5417	0.082*	
C21	0.3831 (6)	0.1739 (6)	0.6093 (4)	0.096 (3)	
H21	0.3275	0.1436	0.6012	0.115*	
C22	0.4222 (9)	0.1586 (7)	0.6560 (4)	0.118 (4)	
H22	0.3929	0.1177	0.6802	0.141*	
C23	0.5037 (8)	0.2019 (7)	0.6685 (3)	0.114 (3)	
H23	0.5299	0.1899	0.7009	0.137*	
C24	0.5477 (5)	0.2642 (6)	0.6326 (3)	0.084 (2)	
H24	0.6028	0.2948	0.6415	0.101*	
S3	0.6482 (5)	0.2367 (6)	0.2460 (2)	0.148 (2)	0.555 (7)
O1	0.6973 (12)	0.2972 (13)	0.2789 (6)	0.205 (6)	0.555 (7)
C25	0.6929 (15)	0.1269 (10)	0.2344 (8)	0.153 (5)	0.555 (7)
H25A	0.7149	0.0982	0.2663	0.230*	0.555 (7)
H25B	0.7428	0.1343	0.2104	0.230*	0.555 (7)
H25C	0.6474	0.0834	0.2195	0.230*	0.555 (7)
C26	0.6108 (16)	0.2920 (16)	0.1935 (6)	0.168 (6)	0.555 (7)
H26A	0.5859	0.3568	0.2023	0.252*	0.555 (7)
H26B	0.5641	0.2513	0.1777	0.252*	0.555 (7)
H26C	0.6605	0.3006	0.1694	0.252*	0.555 (7)
S3A	0.7135 (7)	0.2550 (8)	0.2128 (3)	0.174 (3)	0.445 (7)
O1A	0.7568 (16)	0.3180 (16)	0.2464 (8)	0.219 (6)	0.445 (7)
C25A	0.6772 (18)	0.1491 (12)	0.2385 (10)	0.140 (5)	0.445 (7)
H25D	0.7254	0.1193	0.2588	0.210*	0.445 (7)
H25E	0.6595	0.1034	0.2113	0.210*	0.445 (7)
H25F	0.6256	0.1625	0.2605	0.210*	0.445 (7)
C26A	0.6357 (16)	0.307 (2)	0.1760 (10)	0.176 (7)	0.445 (7)
H26D	0.6588	0.3690	0.1620	0.264*	0.445 (7)
H26E	0.5819	0.3203	0.1963	0.264*	0.445 (7)
H26F	0.6205	0.2620	0.1480	0.264*	0.445 (7)
O2	0.5000	0.034 (2)	0.2500	0.263 (14)*	0.50

### Atomic displacement parameters (Å^2^)

**Table:**

	*U*^11^	*U*^22^	*U*^33^	*U*^12^	*U*^13^	*U*^23^
Pd	0.0372 (3)	0.0535 (4)	0.0320 (3)	0.0035 (3)	−0.0035 (3)	0.0020 (3)
S1	0.0524 (9)	0.0909 (13)	0.0409 (9)	−0.0164 (9)	0.0029 (8)	0.0012 (9)
S2	0.0407 (8)	0.0700 (11)	0.0374 (8)	−0.0005 (7)	−0.0034 (6)	0.0048 (7)
C2	0.044 (3)	0.047 (4)	0.037 (3)	0.004 (3)	0.002 (3)	0.005 (3)
N3	0.044 (3)	0.072 (3)	0.043 (3)	−0.004 (3)	−0.005 (2)	0.004 (3)
N4	0.051 (3)	0.070 (4)	0.037 (3)	−0.010 (3)	−0.003 (2)	0.005 (3)
C5	0.055 (4)	0.073 (5)	0.041 (4)	−0.006 (3)	−0.005 (3)	0.000 (3)
N6	0.070 (4)	0.154 (6)	0.037 (3)	−0.015 (4)	0.001 (3)	0.005 (4)
P1	0.0462 (9)	0.0563 (10)	0.0381 (8)	0.0086 (8)	−0.0024 (7)	0.0037 (8)
C7	0.046 (4)	0.071 (4)	0.040 (3)	0.012 (3)	−0.002 (3)	0.011 (3)
C8	0.063 (5)	0.128 (7)	0.062 (4)	0.024 (5)	−0.009 (4)	−0.018 (5)
C9	0.056 (5)	0.178 (10)	0.087 (6)	0.037 (6)	−0.016 (4)	−0.026 (6)
C10	0.053 (5)	0.178 (10)	0.084 (6)	0.010 (6)	−0.019 (4)	−0.009 (6)
C11	0.078 (6)	0.128 (7)	0.087 (6)	−0.015 (6)	−0.017 (5)	−0.023 (5)
C12	0.054 (4)	0.094 (6)	0.072 (5)	0.006 (4)	−0.013 (4)	−0.010 (4)
C13	0.048 (4)	0.071 (5)	0.041 (4)	0.005 (3)	0.007 (3)	−0.005 (3)
C14	0.059 (4)	0.058 (4)	0.064 (5)	−0.005 (3)	0.005 (4)	−0.003 (4)
C15	0.080 (5)	0.093 (6)	0.056 (4)	−0.010 (5)	0.014 (4)	−0.015 (5)
C16	0.076 (5)	0.069 (6)	0.100 (7)	−0.002 (4)	0.031 (5)	−0.030 (5)
C17	0.092 (6)	0.070 (6)	0.113 (7)	0.031 (5)	0.019 (5)	−0.006 (6)
C18	0.089 (5)	0.068 (5)	0.073 (5)	0.034 (4)	0.002 (4)	0.005 (5)
C19	0.073 (5)	0.048 (4)	0.046 (4)	0.016 (4)	0.007 (4)	0.001 (3)
C20	0.094 (6)	0.052 (4)	0.058 (4)	−0.002 (4)	0.022 (4)	0.002 (4)
C21	0.127 (8)	0.068 (6)	0.093 (6)	−0.024 (5)	0.035 (6)	−0.006 (5)
C22	0.186 (12)	0.069 (6)	0.099 (8)	−0.011 (7)	0.067 (8)	0.012 (6)
C23	0.188 (11)	0.100 (7)	0.054 (5)	0.009 (8)	0.019 (7)	0.027 (5)
C24	0.111 (6)	0.087 (6)	0.055 (5)	0.012 (5)	0.008 (4)	0.013 (4)
S3	0.146 (5)	0.196 (6)	0.104 (4)	0.051 (4)	−0.005 (4)	0.006 (4)
O1	0.245 (14)	0.220 (10)	0.149 (11)	−0.009 (10)	−0.024 (9)	−0.004 (9)
C25	0.122 (11)	0.218 (9)	0.120 (11)	0.086 (9)	−0.060 (9)	−0.007 (8)
C26	0.166 (11)	0.186 (10)	0.151 (10)	0.042 (10)	−0.020 (9)	0.042 (8)
S3A	0.169 (7)	0.230 (7)	0.123 (6)	0.024 (6)	−0.007 (5)	0.011 (5)
O1A	0.216 (14)	0.267 (12)	0.173 (13)	−0.040 (11)	−0.020 (10)	0.008 (10)
C25A	0.139 (11)	0.166 (10)	0.115 (10)	0.080 (8)	−0.025 (9)	−0.014 (8)
C26A	0.188 (14)	0.198 (13)	0.141 (13)	0.019 (11)	−0.003 (10)	0.046 (10)

### Geometric parameters (Å, º)

**Table:**

Pd—P1^i^	2.3363 (15)	C16—H16	0.9300
Pd—P1	2.3364 (15)	C17—C18	1.368 (9)
Pd—S2^i^	2.3407 (14)	C17—H17	0.9300
Pd—S2	2.3407 (14)	C18—H18	0.9300
S1—C5	1.748 (6)	C19—C24	1.377 (8)
S1—C2	1.751 (5)	C19—C20	1.402 (8)
S2—C2	1.736 (5)	C20—C21	1.367 (9)
C2—N3	1.288 (7)	C20—H20	0.9300
N3—N4	1.394 (6)	C21—C22	1.344 (12)
N4—C5	1.284 (7)	C21—H21	0.9300
C5—N6	1.348 (7)	C22—C23	1.362 (12)
N6—H6A	0.904 (10)	C22—H22	0.9300
N6—H6B	0.899 (10)	C23—C24	1.396 (10)
P1—C19	1.807 (6)	C23—H23	0.9300
P1—C13	1.817 (6)	C24—H24	0.9300
P1—C7	1.821 (6)	S3—O1	1.369 (12)
C7—C8	1.367 (8)	S3—C25	1.623 (8)
C7—C12	1.382 (8)	S3—C26	1.632 (7)
C8—C9	1.402 (10)	C25—H25A	0.9600
C8—H8	0.9300	C25—H25B	0.9600
C9—C10	1.324 (11)	C25—H25C	0.9600
C9—H9	0.9300	C26—H26A	0.9600
C10—C11	1.348 (10)	C26—H26B	0.9600
C10—H10	0.9300	C26—H26C	0.9600
C11—C12	1.391 (9)	S3A—O1A	1.358 (12)
C11—H11	0.9300	S3A—C26A	1.633 (8)
C12—H12	0.9300	S3A—C25A	1.640 (8)
C13—C14	1.374 (7)	C25A—H25D	0.9600
C13—C18	1.397 (8)	C25A—H25E	0.9600
C14—C15	1.401 (8)	C25A—H25F	0.9600
C14—H14	0.9300	C26A—H26D	0.9600
C15—C16	1.351 (10)	C26A—H26E	0.9600
C15—H15	0.9300	C26A—H26F	0.9600
C16—C17	1.348 (10)		
			
P1^i^—Pd—P1	180.0	C16—C17—C18	120.1 (7)
P1^i^—Pd—S2^i^	94.12 (5)	C16—C17—H17	119.9
P1—Pd—S2^i^	85.88 (5)	C18—C17—H17	119.9
P1^i^—Pd—S2	85.89 (5)	C17—C18—C13	121.1 (7)
P1—Pd—S2	94.12 (5)	C17—C18—H18	119.4
S2^i^—Pd—S2	180.0	C13—C18—H18	119.4
C5—S1—C2	86.6 (3)	C24—C19—C20	116.7 (6)
C2—S2—Pd	103.87 (19)	C24—C19—P1	124.2 (6)
N3—C2—S2	127.2 (4)	C20—C19—P1	119.1 (5)
N3—C2—S1	113.7 (4)	C21—C20—C19	122.4 (7)
S2—C2—S1	119.0 (3)	C21—C20—H20	118.8
C2—N3—N4	112.7 (5)	C19—C20—H20	118.8
C5—N4—N3	113.3 (5)	C22—C21—C20	119.2 (9)
N4—C5—N6	125.0 (5)	C22—C21—H21	120.4
N4—C5—S1	113.6 (4)	C20—C21—H21	120.4
N6—C5—S1	121.4 (5)	C21—C22—C23	121.4 (9)
C5—N6—H6A	107.1 (13)	C21—C22—H22	119.3
C5—N6—H6B	107.0 (13)	C23—C22—H22	119.3
H6A—N6—H6B	116.7 (19)	C22—C23—C24	119.7 (9)
C19—P1—C13	99.9 (3)	C22—C23—H23	120.2
C19—P1—C7	105.3 (3)	C24—C23—H23	120.2
C13—P1—C7	106.6 (3)	C19—C24—C23	120.7 (8)
C19—P1—Pd	121.6 (2)	C19—C24—H24	119.7
C13—P1—Pd	113.4 (2)	C23—C24—H24	119.7
C7—P1—Pd	108.7 (2)	O1—S3—C25	115.4 (8)
C8—C7—C12	118.7 (6)	O1—S3—C26	115.0 (9)
C8—C7—P1	123.9 (6)	C25—S3—C26	112.7 (9)
C12—C7—P1	117.4 (5)	S3—C25—H25A	109.5
C7—C8—C9	120.3 (7)	S3—C25—H25B	109.5
C7—C8—H8	119.8	H25A—C25—H25B	109.5
C9—C8—H8	119.8	S3—C25—H25C	109.5
C10—C9—C8	120.4 (8)	H25A—C25—H25C	109.5
C10—C9—H9	119.8	H25B—C25—H25C	109.5
C8—C9—H9	119.8	S3—C26—H26A	109.5
C9—C10—C11	120.2 (8)	S3—C26—H26B	109.5
C9—C10—H10	119.9	H26A—C26—H26B	109.5
C11—C10—H10	119.9	S3—C26—H26C	109.5
C10—C11—C12	121.4 (8)	H26A—C26—H26C	109.5
C10—C11—H11	119.3	H26B—C26—H26C	109.5
C12—C11—H11	119.3	O1A—S3A—C26A	115.7 (10)
C7—C12—C11	119.0 (7)	O1A—S3A—C25A	114.9 (9)
C7—C12—H12	120.5	C26A—S3A—C25A	111.7 (10)
C11—C12—H12	120.5	S3A—C25A—H25D	109.5
C14—C13—C18	118.2 (6)	S3A—C25A—H25E	109.5
C14—C13—P1	120.2 (5)	H25D—C25A—H25E	109.5
C18—C13—P1	121.5 (5)	S3A—C25A—H25F	109.5
C13—C14—C15	119.1 (6)	H25D—C25A—H25F	109.5
C13—C14—H14	120.4	H25E—C25A—H25F	109.5
C15—C14—H14	120.4	S3A—C26A—H26D	109.5
C16—C15—C14	121.1 (7)	S3A—C26A—H26E	109.5
C16—C15—H15	119.5	H26D—C26A—H26E	109.5
C14—C15—H15	119.5	S3A—C26A—H26F	109.5
C17—C16—C15	120.3 (7)	H26D—C26A—H26F	109.5
C17—C16—H16	119.8	H26E—C26A—H26F	109.5
C15—C16—H16	119.8		

Symmetry code: (i) −*x*+1, −*y*+1, −*z*+1.

### Hydrogen-bond geometry (Å, º)

**Table:**

*D*—H···*A*	*D*—H	H···*A*	*D*···*A*	*D*—H···*A*
N6—H6*A*···N4^ii^	0.90 (1)	2.12 (2)	2.986 (7)	160 (5)

Symmetry code: (ii) −*x*+1, *y*, −*z*+3/2.
